# Structural and Functional Properties of the Capsid Protein of Dengue and Related *Flavivirus*

**DOI:** 10.3390/ijms20163870

**Published:** 2019-08-08

**Authors:** André F. Faustino, Ana S. Martins, Nina Karguth, Vanessa Artilheiro, Francisco J. Enguita, Joana C. Ricardo, Nuno C. Santos, Ivo C. Martins

**Affiliations:** 1 Instituto de Medicina Molecular, Faculdade de Medicina, Universidade de Lisboa, Av. Prof. Egas Moniz, 1649-028 Lisbon, Portugal; 2 Centro de Química-Física Molecular, Instituto Superior Técnico, Universidade de Lisboa, 1049-001 Lisbon, Portugal

**Keywords:** Dengue virus (DENV), capsid protein (C protein), *Flavivirus*, intrinsically disordered protein (IDP), protein–RNA interactions, protein–host lipid systems interaction, circular dichroism, time-resolved fluorescence anisotropy

## Abstract

Dengue, West Nile and Zika, closely related viruses of the Flaviviridae family, are an increasing global threat, due to the expansion of their mosquito vectors. They present a very similar viral particle with an outer lipid bilayer containing two viral proteins and, within it, the nucleocapsid core. This core is composed by the viral RNA complexed with multiple copies of the capsid protein, a crucial structural protein that mediates not only viral assembly, but also encapsidation, by interacting with host lipid systems. The capsid is a homodimeric protein that contains a disordered N-terminal region, an intermediate flexible fold section and a very stable conserved fold region. Since a better understanding of its structure can give light into its biological activity, here, first, we compared and analyzed relevant mosquito-borne *Flavivirus* capsid protein sequences and their predicted structures. Then, we studied the alternative conformations enabled by the N-terminal region. Finally, using dengue virus capsid protein as main model, we correlated the protein size, thermal stability and function with its structure/dynamics features. The findings suggest that the capsid protein interaction with host lipid systems leads to minor allosteric changes that may modulate the specific binding of the protein to the viral RNA. Such mechanism can be targeted in future drug development strategies, namely by using improved versions of pep14-23, a dengue virus capsid protein peptide inhibitor, previously developed by us. Such knowledge can yield promising advances against Zika, dengue and closely related *Flavivirus*.

## 1. Introduction

Viral hemorrhagic fever is a global problem, with most cases due to dengue virus (DENV), which originates over 390 million infections per year worldwide, being a major socio-economic burden, mainly for tropical and subtropical developing countries [1]. A working vaccine was registered in Mexico in December 2015, approved for official use in some endemic regions of Latin America and Asia and, as of October 2018, also in Europe [2,3,4]. However, this vaccine is not 100% effective against all DENV serotypes. Thus, research into new prophylactics is still ongoing, with a new vaccine proposed recently being now in phase 3 clinical trials [5]. In spite of these recent developments, fully effective prophylactics approaches are lacking and there are no effective therapies. This is in part, due to a poor understanding of key steps of the viral life cycle.

There are four dengue serotypes occurring: DENV-1, DENV-2, DENV-3 and DENV-4 [6]. Here, if not otherwise indicated, DENV refers to DENV-2. DENV is a member of the *Flavivirus* genus, part of the Flaviviridae family, a genus which comprises 53 viral species [6]. Many of these are important human pathogens as well, such as hepatitis C (HCV), tick-borne encephalitis (TBEV), yellow fever (YFV), West Nile (WNV) and Zika (ZIKV) viruses [6,7,8,9]. Flaviviridae are single-stranded positive-sense RNA viruses with approximately 11 kb, containing a single open reading frame [10]. Using the host cell translation machinery, the *Flavivirus* RNA genome is translated into a polyprotein that is co- and post-translationally cleaved by cellular and viral proteases into three structural proteins and seven non-structural proteins [10]. Structural proteins are named as such since they are present in the mature virion structure [11]. Nevertheless, they may also have non-structural roles, such as the capsid (C) protein. This is a structural protein that also mediates viral assembly and encapsidation, crucial steps of the viral life cycle. Given the C protein key roles, it is the focus of this work and will be described in detail below.

DENV C contains 100 amino acid residues, which form an homodimer with an intrinsically disordered protein (IDP) region in the N-terminal followed by four α-helices, α1 to α4, per monomer [12]. Overall, the main structural/dynamics regions consist of the disordered N-terminal, a short flexible intermediate fold and, finally, a large conserved fold region, which greatly stabilizes the protein homodimer structure [12,13,14,15,16]. The C protein has an asymmetric charge distribution: one side of the dimer contains a hydrophobic pocket (α2–α2′ interface), responsible for, alongside the disordered N-terminal, the binding to host lipid droplets (LDs) [12,13,14,15,16]. The other is the positively charged C-terminal side (α4–α4′ interface), proposed to mediate the C protein binding to the viral RNA [12]. It is noteworthy that several transient conformations for DENV C N-terminal were proposed, which may help modulate DENV C interaction with host lipid systems, via an autoinhibition mechanism [15].

DENV infection affects the host lipid metabolism, increasing host intracellular LDs and unbalancing plasma lipoprotein levels and composition [17,18,19]. Importantly, DENV C binds LDs, an interaction essential for viral replication [18,20]. DENV C-LDs binding requires potassium ions, the LDs surface protein perilipin 3 (PLIN3) and involves specific amino acid residues of DENV C α2–α2′ helical hydrophobic core and of the N-terminal [14,20]. This knowledge led us to design pep14-23, a patented peptide, based on a *Flavivirus* C protein conserved N-terminal motif. We then established that pep14-23 inhibits DENV C-LDs binding [14], acquiring α-helical structure in the presence of anionic phospholipids [15]. Moreover, we also found that DENV C binds specifically to very low-density lipoproteins (VLDL), requiring K^+^ ions and a specific VLDL surface protein, apolipoprotein E (APOE), being also inhibited by pep14-23 [21]. This is analogous to DENV C-LDs interaction. The similarities between APOE and PLIN3 further reinforce this, suggesting a common mechanism [22]. The role of LDs in *Flavivirus* infection is well known and has been recently reviewed [14,18,20,23,24,25]. Given that, pep14-23 is an excellent drug development lead. Further developments require a better understanding of the function of the C protein of dengue and of *Flavivirus* in general.

Therefore, here, we seek to contribute to understand the C proteins biological activity, with a special focus on DENV C. Briefly, we studied DENV C structure-activity relationship in the context of similar and highly homologous mosquito-borne *Flavivirus* C proteins. Our findings shed light into the structure-function relationship behind the C protein biological roles, which may contribute to future therapeutic approaches against DENV and closely related *Flavivirus*.

## 2. Results

### 2.1. Analysis of Amino Acid Sequence Conservation Among Flavivirus C proteins

**Figure 1:**
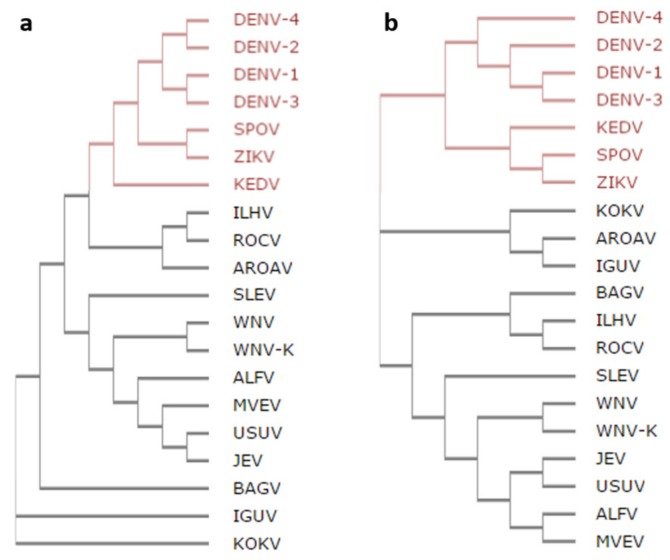
*Flavivirus* phylogenetic trees. Phylogenetic trees of (**a**) *Flavivirus* C proteins, highlighting in red the viruses with the C protein most similar to dengue virus (DENV) C (Spondweni group viruses (ZIKV), Spondweni virus (SPOV) and Kedougou virus (KEDV)) and of the (**b**) entire viral polyproteins of the same *Flavivirus*. Overall, despite some differences, the same general clusters are seen regardless of the clustering being based on the polyprotein or the capsid protein.

A phylogenetic analysis of the *Flavivirus* C protein and the polyprotein amino acid residue sequences reveals if the C protein is an indicator of phylogenetic similarity (Figure 1). C proteins of Spondweni group viruses, i.e., ZIKV, Spondweni virus (SPOV) and Kedougou virus (KEDV), cluster together, being the most similar to DENV (Figure 1a). Another cluster corresponds to mosquito-borne encephalitis-causing *Flavivirus*: Saint Louis encephalitis (SLEV), WNV, WNV serotype Kunjin (WNV-K), Alfuy (ALFV), Murray Valley encephalitis (MVEV), Usutu (USUV) and Japanese encephalitis (JEV) viruses. The *Flavivirus* polyproteins sequences show similar clusters (Figure 1b). As such, the C protein is a good indicator of viral genetic similarity. Thus, we investigated the C protein amino acid sequences, seeking common patterns relevant to biological activity.

**Figure 2:**
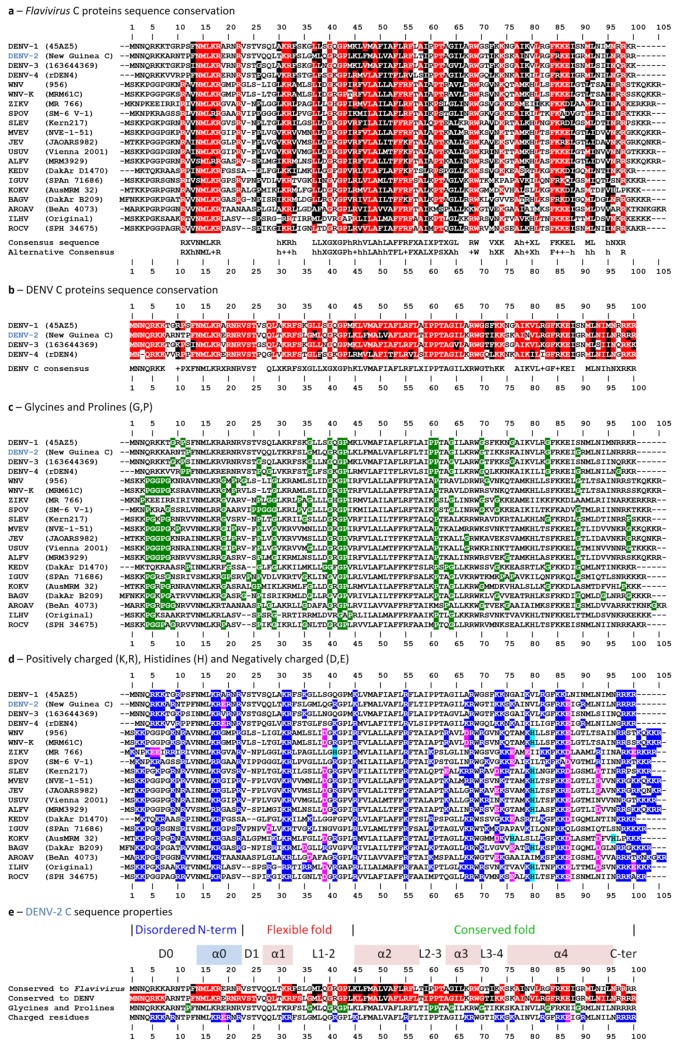
*Flavivirus* C proteins amino acid residues sequence conservation. (**a**) Mosquito-borne *Flavivirus* C protein are 55% conserved, with residues being considered conserved if, in a given position, more than 15 are equal (red) or stereochemically similar (black). (**b**) Conservation between DENV serotypes is 80%, with the same criteria as in (**a**). (**c**) Structure-breaking residues G and P (green). (**d**) Charged residues: dark blue for positively charged residues (K and R), light blue for H, and magenta for negatively charged residues (D or E). (**e**) Overall conserved regions of *Flavivirus* C proteins: the disordered N-terminal and the conserved fold are clearly conserved in terms of charged and G/P amino acids. In contrast, the flexible fold region allows higher variability. Thus, its main role seems to be to connect the disordered N-terminal and the conserved fold regions, and to enable alternative conformations. DENV C serotype 2 is highlighted in blue, with amino acid residues numbered according to its sequence. Amino acid residues are numbered according to the consensus, coinciding with DENV-2 residues numbers. The viruses’ full designation is found in the abbreviations section.

The amino acid residues sequences of the *Flavivirus* C proteins identified above were analyzed in the context of the three main regions identified in DENV C sequence, *i.e.*, the conserved fold region, the flexible fold region and the N-terminal IDP region (Figure 2). This was done for all mosquito-borne *Flavivirus* relevant for human diseases (Figure 2a), as well as for the four main DENV C serotypes (Figure 2b). For this, the 16 mosquito-borne *Flavivirus* and the 4 DENV serotypes amino acid sequence of the C protein are jointly aligned. In agreement with previous work [12,14], five conserved motifs are found in the mosquito-borne *Flavivirus* C proteins and deserve attention, namely: the N-terminal conserved ^13^hNML+R^18^; ^40^GXGP^43^ in loop L1-2; ^44^h+hhLAhhAFF+F^56^ in α2 helix; ^68^RW^69^ of α3 helix; and, finally, the ^84^F++–h^88^ motif from α4 (with ‘h’, ‘+’ and ‘–’ representing hydrophobic, positively charged and negatively charged residues, respectively). Between residues 70–100, other motifs, not previously reported and containing hydrophobic and positively charged residues, are visible. Moreover, amino acid residues G and P, that can break the continuity of α-helices, are conserved in specific positions of the protein, especially in the disordered N-terminal and the flexible fold regions (Figure 2c). Charged residues are also conserved in specific locations. They are mostly in the conserved fold region, especially after position 95 (Figure 2d). Overall, the disordered N-terminal and the flexible fold regions, when compared with the conserved fold region, have an average of, respectively, 10 versus 4 G and P residues (Figure 2c), green, 10 versus 15 K and R residues (Figure 2d), blue, and 1 versus 2 D and E residues (Figure 2d), magenta.

Several motifs in the *Flavivirus* C protein sequences can be identified. These represent the main sections of the protein, conserved during evolution as these must be crucial to protein function (Figure 2e). The N-terminal region, although disordered, is highly conserved, in terms of charged amino acid and G/P residues. The flexible fold section allows greater variability, in line with previous reports by us and others, suggesting that it can adopt several conformations [15].

### 2.2. Analysis of the Flavirus C Protein Sequences Hydrophobicity and Secondary Structure Propensity

**Figure 3:**
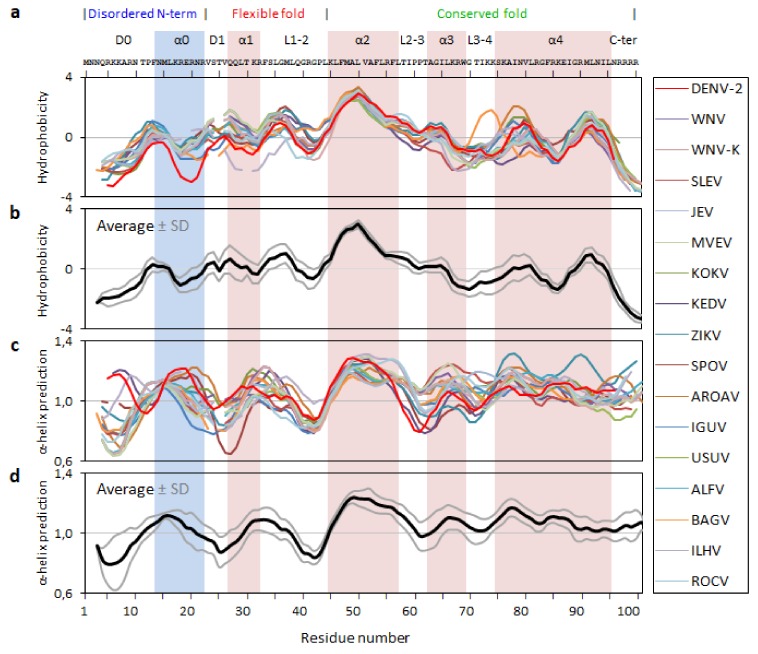
*Flavivirus* C proteins hydrophobicity and secondary structure predictions. (**a**) Hydrophobicity predictions and (**b**) respective average (black line) ± standard deviation, SD (gray lines). (**c**) α-helical secondary structure predictions and (**d**) respective average (black line) ± SD (gray lines). Amino acid residues are numbered according to the consensus, coinciding with DENV 2 residues numbers.

Hydrophobicity and α-helical propensity predictions were performed as previously reported [15], using the Kite-Doolittle [26] and the Deleage-Roux [27] scales on ProtScale server, respectively, for the 16 mosquito-borne *Flavivirus* C proteins analyzed (Figure 3). The hydrophobicity scale ranges from −4.5, for highly polar amino acids (hydrophilic), to 4.5, for highly hydrophobic amino acid residues [26]. Therefore, when plotting the average values for each amino acid residue of the *Flavivirus* C sequences, negative local minima and positive local maxima indicate, respectively, hydrophilic and hydrophobic regions (Figure 3a,b). All proteins display a similar profile even in the N-terminal and flexible fold regions despite the slightly higher amino acid residues variability (Figure 2). The α0 domain, homologous to pep14-23, is amphipathic, with average values near 0. In the flexible fold region, which is mostly amphipathic too, there is a peak of hydrophobicity between residues 30 and 40, possibly explaining its intermediate structure/dynamics behavior [13,14]. Some peaks of hydrophobicity are observed in the α3 and α4 domains, with the most hydrophobic domain being α2, as expected from the sequence analysis (Figure 2) and from the literature [12,14,18].

For α-helical predictions secondary structure is highly probable above a threshold of 1.0 [27]. *Flavivirus* C proteins secondary structure predictions correlate well with the known secondary structure of DENV C (Figure 2e) [12]. Such agreement supports the concept of a transient α0 occurring for these proteins, as hypothesized earlier [15]. Roughly, between positions 12 to 20, occurs a disordered region with high tendency to acquire α-helical secondary structure. Importantly, the values of the predictions are similar and the same tendencies are found in all proteins, with peaks and valleys co-localizing (Figure 3). Along with data from the last subsection, these results strengthen the idea that *Flavivirus* C proteins have similar structure and dynamics properties.

### 2.3. Analysis of the Flavivirus C Protein Tertiary Structure Propensity

**Figure 4:**
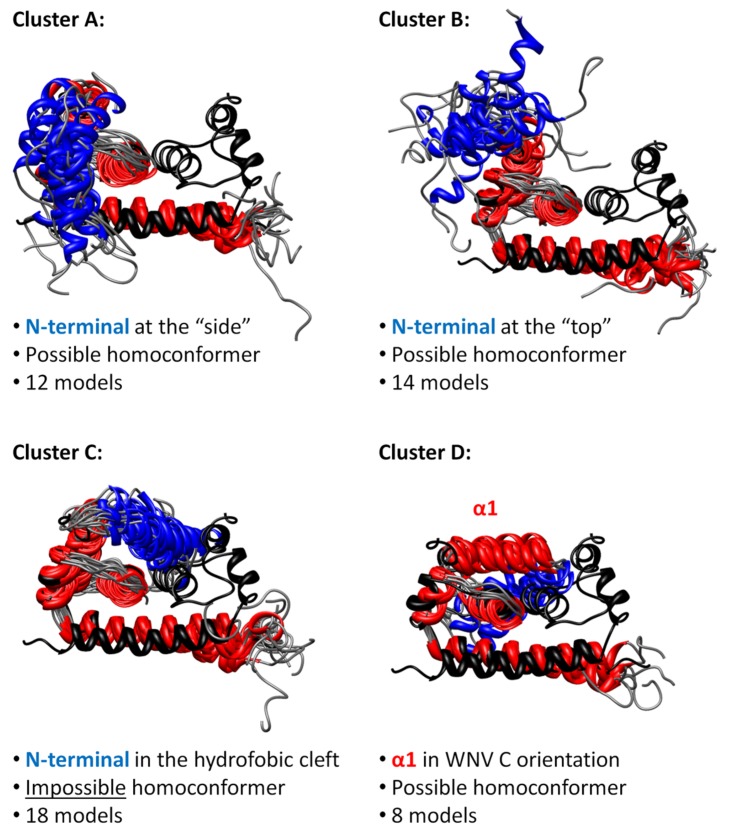
*Flavivirus* C proteins tertiary structure predictions, organized into four conformational clusters. The *Flavivirus* C proteins conformations predicted by I-TASSER are superimposed with DENV C experimental homodimer structure (black). Amino acid residues of the N-terminal region in α-helix conformation are in blue, the other α-helices in red and the loops in gray. From the 80 conformers, 52 can be clustered by similarity of conformations, from cluster A to D. Clusters A, B and C have the α1 helix in the DENV C experimentally determined conformation (Protein Data Bank (PDB) ID: 1R6R [12]). In cluster D the α1 is in West Nile Virus (WNV) C and ZIKV C conformation (PDB IDs: 1SFK [31] and 5YGH [25], respectively). The closed autoinhibitory conformation of cluster C seems the most probable, having the highest number of models. Although unlikely given their transient unstable nature, N-terminal IDP regions may interact with each other. Table 1 specifies each cluster composition.

*Flavivirus* C proteins tertiary structure was then investigated, complementing the α-helical predictions, to help understanding the disordered N-terminal region role(s). Following previous work [15], I-TASSER [28,29,30] was used to predict tertiary structures for the 16 closely related mosquito-borne *Flavivirus* C proteins (Figure 4). Eighty monomer conformations were obtained (several for each sequence) and superimposed with the DENV C homodimer partial structure deposited at the Protein Data Bank (PDB) and obtained via nuclear magnetic resonance (NMR) spectroscopy (PDB ID: 1R6R). Noteworthy, DENV [12,16], WNV [31] and ZIKV [25] C proteins form homodimers, stabilized by hydrophobic and electrostatic interactions involving their conserved fold region [12,13,14,25,31,32,33]. Since this is the most conserved region of *Flavivirus* C proteins sequences (Figure 2), a homodimer is thus not only a stable conformational arrangement, but also likely to occur. Thus, as 28 conformers had more than 5 backbone clashes with the other monomer when superimposed in a homodimer structure (not allowing a viable homodimer), those conformers were discarded Table 1. The remaining 52 *Flavivirus* C proteins conformational models were analyzed, while superimposed with DENV C homodimer (PDB ID: 1R6R, model 21 [12]). These were then grouped into four clusters by visual inspection of their similarity (Figure 4).

Most sequences have a conformer in each cluster (Figure 1 and Table 1). In cluster A, some N-terminal amino acid residues are close to α4–α4′ and may interact with RNA, namely the positively charged residues. Cluster B has the most scattered conformers, with the N-terminal region at the “top”, not interacting with other protein regions, resembling a transition between more ordered states. In cluster C, the N-terminal region is in an autoinhibitory conformation, blocking the access to the α1–α2–α2′–α1′ region, as previously suggested by us for DENV C [15]. 18 conformer models are predicted in this closed conformation with, at least, one model from most of the C proteins tested (except JEV C and ZIKV C; see Table 1). Therefore, it can occur in most *Flavivirus* C proteins. As for cluster D conformation, the α1 helix is in the conformation of WNV [14,31] and ZIKV [25] C experimental structures, an arrangement not previously reported for DENV C [15]. This closed conformation also involves the N-terminal region and α1 domain, and partially blocks the α2–α2′ hydrophobic cleft (or totally blocks it, when both monomers are in the same conformation). Importantly, both cluster C and D are closed conformations, supporting the autoinhibition hypothesis.

Dimers with A or B conformers in one monomer enable the simultaneous co-existence of all other conformers (A to D) on the other monomer. The C conformer neither permits the existence of C-C′ homoconformers (i.e., both monomers in the same conformation) nor the heteroconformers of C-D′ and D-C′. Despite that, D-D′ homoconformers are allowed, similarly to the conformation that WNV C adopts in the crystal form [31]. Moreover, to go from cluster A to cluster C or D, the N-terminal region should pass by cluster B. These constraints suggest a path for transitions between conformations, discussed ahead. Overall, the autoinhibition hypothesis proposed for DENV C [15] is supported and such conformation can occur in other *Flavivirus* C proteins.

### 2.4. Analysis of Dengue Virus (DENV) C Protein Rotational Correlation Time

**Figure 5:**
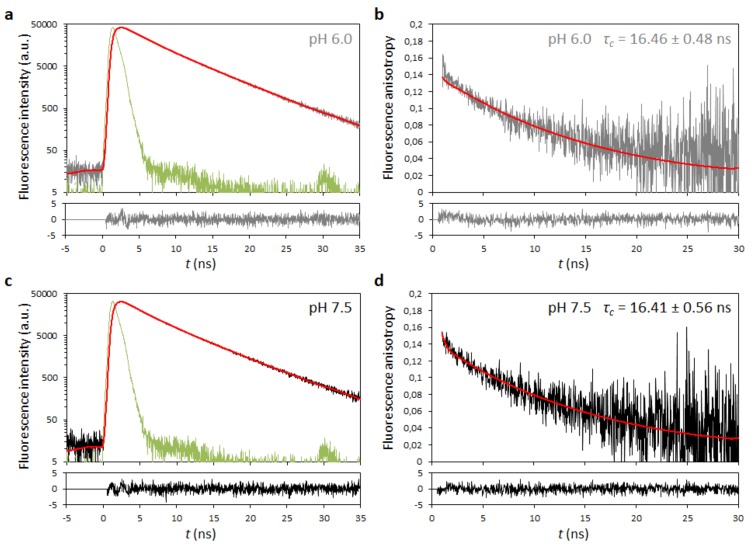
DENV C time-resolved fluorescence anisotropy. Time-resolved fluorescence decay at pH (**a**) 6.0 and (**c**) 7.5, with the corresponding anisotropy decays at pH (**b**) 6.0 and (**d**) 7.5. Fluorescence and anisotropy decays at both pH values are similar (gray and black decays, respectively). Fitting of experimental data (red) took into account the instrument response function (IRF; in green) and the corresponding residuals distribution, displayed below each graph. The equations used for fitting are presented on the Methods Equations (5) and (8). The parameters obtained are shown in Table 2.

Given the close similarities between *Flavivirus* C proteins (Figure 1, Figure 2, Figure 3 and Figure 4), DENV C can be used as a general model for them. Hence, we proceeded to determine DENV C overall rotational correlation time (*τ_c_*), taking advantage of the tryptophan residue in position 69 (W69) intrinsic fluorescence. Our computational data support three main structure/dynamics regions, including a disordered N-terminal region, which would increase its expected apparent size (as it would not be globular and folded), a property detectable by such an approach. Upon testing molecules in aqueous solution and at room temperature, fluorescence lifetimes are usually in the ns timescale, and the fluorescence decays are sensitive to the anisotropy of the fluorophore, which depends on its *τ_c_* (vd. Equations (1)–(8), describing these relations, in the Methods section [34,35]). Thus, the time-resolved fluorescence decay of DENV C W69 and the corresponding anisotropy decay were determined, both at pH 6.0 and 7.5 (Figure 5).

Time-resolved fluorescence anisotropy decays at both pH values are similar (Figure 5b,d). Fluorescence lifetime components (*τ*_1_, *τ*_2_ and *τ*_3_) were obtained from the intensity decays Equations (2)–(6) [34,35], with a triple-exponential retrieving the best fit (Figure 5a,c). Fitting the data retrieves similar values Table 2 for *τ*_1_, *τ*_2_ and *τ*_3_, and corresponding weights (α_1_, α_2_ and α_3_ pre-exponential factors, respectively). For accurate calculation of *τ_c_*, the condition *τ_c_* < 3 × *τ*_3_ must occur [34,35]. Since *τ*_3_ values were ~6.4 ns (with a significant weight α_3_ of ~0.42), this means that, at both pH values, we could measure *τ_c_* values up to a limit of ~19 ns. In both pH conditions, the *τ_c_* measured was 16.4 ± 0.5 ns at 22 °C, within the limit and higher than expected for a purely globular protein of DENV C size, as predicted [13]. 

Rossi et al. [36] correlated the *τ_c_* of 16 globular proteins at 20 °C with their molecular weight (MW in kDa), based on NMR data, leading to the relation: *τ_c_* ≈ 0.6 MW. Assuming DENV C as a 23.5 kDa fully globular homodimer and correcting for the temperature (*T*) and viscosity (*η*) [37], the *τ_c_* predicted is 12.0 ns. However, the correlational time must be slightly higher, as the protein will be partially unfolded and disordered (in the N-terminal). Jones et al. [16] measured a *τ_c_* of 13 ns at 27 °C, by NMR, which with the corrections from Equation (10) [37], corresponds to 13.4 ns at 25 °C. Given DENV C size, this implies that the protein is not globular, in line with current knowledge of DENV C structure and dynamics [12,13,14,15,16]. Fluorescence anisotropy supports an even more open and partially disordered DENV C structure, given the *τ_c_* value of 15.2 ± 0.5 ns at 25 °C Table 3, in line with in silico data (Figure 1, Figure 2, Figure 3 and Figure 4).

### 2.5. Analysis of DENV C Conformational Stability

**Figure 6:**
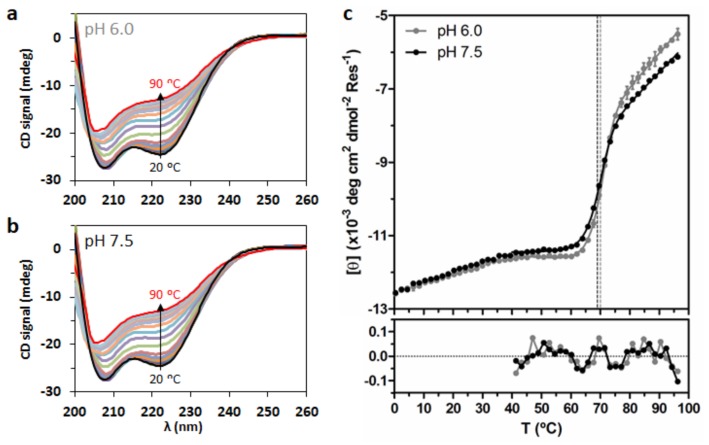
DENV C temperature denaturation followed via circular dichroism (CD) spectroscopy. CD spectra of DENV C, between 20 and 90 °C, at pH (**a**) 6.0 and (**b**) 7.5. For the sake of simplicity, the spectra from 0 to 18 °C and from 92 to 96 °C are not displayed, as they are similar to the 20 °C and the 90 °C spectra, respectively. (**c**) Mean residue molar ellipticity at 222 nm, [θ], as a function of temperature (dots) for pH 6.0 (gray) and 7.5 (black), between 0 and 96 °C. Lines correspond to the fitting of Equation (21) (combined with Equations (20), (22), (24) and (28)). Vertical dashed lines represent experimentally observed *T*_m_, colored according to pH. Error bars represent SD, from three independent experiments. Residuals are shown below the graph, being lower than SD.

Circular dichroism (CD) spectroscopy was used to study DENV C secondary structure, via its thermal denaturation in solution from 0 to 96 °C, at pH 6.0 and 7.5 (2 °C steps, Figure 6). At both pH values, the α-helical structure is partially lost upon increasing temperature (Figure 6a,b). However, even at 96 °C, the protein does no become completely random coil, as seen from the spectrum shape and its high ellipticity at 222 nm (Figure 6c). Plotting the mean residue molar ellipticity at 222 nm, [θ], as a function of temperature, *T*, reveals a transition at ~70 °C at both pH (Figure 6c). 

DENV C does not display a typical unfolding profile, as the denaturation curves do not reach a flat plateau. Still, ellipticity data were successfully fitted to a denaturation curve (Figure 6c), assuming a homodimer with one-step denaturation [32]. Briefly, Equation (21) was combined with Equations (20), (22) and (24) and fitted to the data. This allows to obtain the thermodynamic parameters of DENV C unfolding Table 4, namely the melting temperature ($T_{m}^{{}^{\circ}}$), the enthalpy variation at $T_{m}^{{}^{\circ}}$ ($\Delta H^{{}^{\circ}}{}_{T_{m}^{{}^{\circ}}}$) and the entropy variation at $T_{m}^{{}^{\circ}}$ ($\Delta S^{{}^{\circ}}{}_{T_{m}^{{}^{\circ}}}$), with all parameters at standard thermodynamics conditions (symbolized by ‘°’). Equation (28) was then used to calculate the melting temperature ($T_{m}$) at the actual $\left[P_{m}\right]$ (instead of the value at $\left[P\right]$ = 1 M, details in the Methods). Despite small differences, the parameters obtained are not significantly different between pH values Table 4. A small but consistent variation of the CD spectra between 0 and 40 °C is observable, implying: (i) a conformational equilibrium with temperature and/or (ii) some flexibility of the structure and/or (iii) a transition between alternative conformations. This temperature range covers the physiological conditions of both mosquitoes (20 to 40 °C, depending on the environment) and humans (36 to 40 °C). DENV C can continuously transition between conformations as temperature varies, in line with the previously hypothesized conformational equilibrium [15]. As temperature increases, the disordered conformations become more abundant but only a partial loss of structure is seen. This indicates that the C protein conserved region is thermodynamically stable. Similar observations are expected for other *Flavivirus* C proteins.

## 3. Discussion

*Flavivirus* C proteins are known to have similar sequences and structure [12,13,14,15,16,25,31]. Here, we go further by examining common features at different structural levels, complemented with data on DENV C size and thermodynamic stability. The phylogenetic analysis of the C proteins and the polyproteins (Figure 1) shows that the former is a marker of *Flavivirus* evolution. There are several conserved motifs, highlighted in previous studies with 16 *Flavivirus* [12,14]. The work is now expanded to include the four DENV serotypes (Figure 2). When these 20 *Flavivirus* C amino acid sequences, with between 96 and 107 amino acid residues each, are jointly analyzed, it is clear that 55% of the residues are conserved or stereochemically similar (Figure 2a). About 80% of amino acid residues are equal or similar and, thus, conserved among the four DENV C serotypes (Figure 2b). From the five major conserved motifs, four are known to be involved in dimer stabilization [14]: the ^40^GXGP^43^ motif at loop L1-2, that marks the transition from the flexible to the conserved fold region [14]; the ^68^RW^69^ at α3 forms an hydrophobic pocket that accommodates the W69 side chain involving residues from α2, α3 and α4 [12,32]; and, the ^44^h+hhLAhhAFF+F^56^ and ^84^F++–h^88^ motifs, respectively from α2 and α4 helices, maintain the homodimer structure both via the α2–α2′ hydrophobic interaction and via the salt bridges of residues [RK]^45^ and [RK]^55′^ with [ED]^87^ [12,14,32]. *Flavivirus* C proteins must have similarly sized secondary structure domains, since G/P are in the same positions and these amino acid residues tend to break the secondary structure (Figure 2c). Charged residues are also conserved (Figure 2d), which makes sense as charges would promote the interaction of the C protein with the negatively charged host lipid systems [12,14,20,21,22] and the viral RNA [12]. C proteins have a common homodimer conserved fold region (roughly, residues 45–100), as observed for DENV, WNV and ZIKV C structures [12,14,25,31]. Conserved motifs are summarized in (Figure 2e).

The above explains the C proteins similar hydrophobic and α-helix propensities (Figure 3). The conserved motif ^13^hNML+R^18^, at the N-terminal region, and the α2–α2′ hydrophobic cleft are of particular importance for DENV C interaction with LDs and VLDL [14,20,21,22,38]. Mutations in specific residues of DENV C α2–α2′ and α4–α4′ also impair RNA binding. Likewise, ZIKV C also accumulates on LDs surface, with specific mutations on this protein disrupting the association [25]. ZIKV C also binds single-stranded and double-stranded RNAs [25], with, as for DENV C, the high positively charged residues density prompting the binding to LDs and RNA [12,39,40]. Given the match at the level of N-terminal α-helical propensity and α2–α2′ hydrophobicity (Figure 3), the C proteins may all be self-regulated by an autoinhibition mechanism, as proposed for DENV C [15].

The autoinhibition hypothesis is corroborated by the quaternary structure analysis (Figure 4); Table 1. Two clusters, C and D, are autoinhibited conformations. Importantly, cluster D α1 aligns with WNV C [14,31] and ZIKV C [25]. Moreover, if two monomers are in a D conformation (D–D′ homoconformer), the dimer α2–α2′ region is totally inaccessible. Cluster C does not allow a C–C′ homoconformer nor a C–D heteroconformer, imposing restrictions to the simultaneous transitions that are possible between A, B, C and D, as homodimer. The interaction between N-terminal regions within a dimer may be considered. Nonetheless, the disordered nature and high density of positively charged amino acid residues will mostly favor the repulsion between these IDP regions.

It is important to look at the clusters (Figure 4), while considering the number of positively charged residues (Figure 2) in the disordered N-terminal and flexible fold (10 K and R residues) versus those in the conserved fold (15 K and R). The charge distribution in some arrangements implies that the disordered N-terminal is at least in theory able to bind the viral RNA [39,40]. Such binding would be governed by the N-terminal region cationic amino acid residues [41,42]. Here, the structure predictions reveal that, indeed, the first 12 N-terminal residues can locate near α4–α4′ Cluster A (Figure 4), the most likely RNA binding site [12,39,40]. Furthermore, binding to RNA via the C-terminal α4–α4′ interface may be favored by a previous or simultaneous interaction of the protein with host LDs via the N-terminal region and α2–α2′ interface. Access to α2–α2′ (controlled by the N-terminal region) would modulate the interaction (Figure 4) and, thus, viral assembly. In agreement, the binding of the related hepatitis C virus core protein (homologous to DENV C) to host LDs is what enables efficient viral assembly [43]. Thus, the C protein disordered N-terminal would be critical to protein function, enabling crucial structural and functional roles.

To evaluate this, we used DENV C as a model system, measuring its *τ_c_* value by time-resolved fluorescence anisotropy (Figure 5) and its thermal stability by CD spectroscopy (Figure 6), at pH 6.0 and 7.5 (within the usual pH range of its biological microenvironment). A similar *τ_c_*, 15.2 ± 0.5 ns, is obtained at both pH values (Figure 5; Table 2 and Table 3), in line with previous work [13]. DENV C maintains its homodimer structure and dynamics behavior between pH 6.0 and 7.5. The *τ_c_* value and respective size are higher than expected, due to the N-terminal disordered nature.

Regarding DENV C thermodynamic stability (Figure 6, Table 4), the protein *T*_m_ is ~70 °C at both pH values. These denaturation parameters are in line with other authors, as a chemically synthesized DENV C 21–100 fragment (without most of the disordered N-terminal region) displays a *T*_m_ = 71.6 °C [32]. DENV C high thermal stability in physiological conditions is likely due to the large hydrophobic area that is shared by the two monomers [12], but also to the W69 stabilizing interactions and, as experimentally observed [32], the formation of salt bridges (residues K45 and R55′ with E87). As structure/dynamics properties are conserved among *Flavivirus* C proteins (Figure 2, Figure 3 and Figure 4), these observations can probably be generalized for all these proteins.

**Figure 7:**
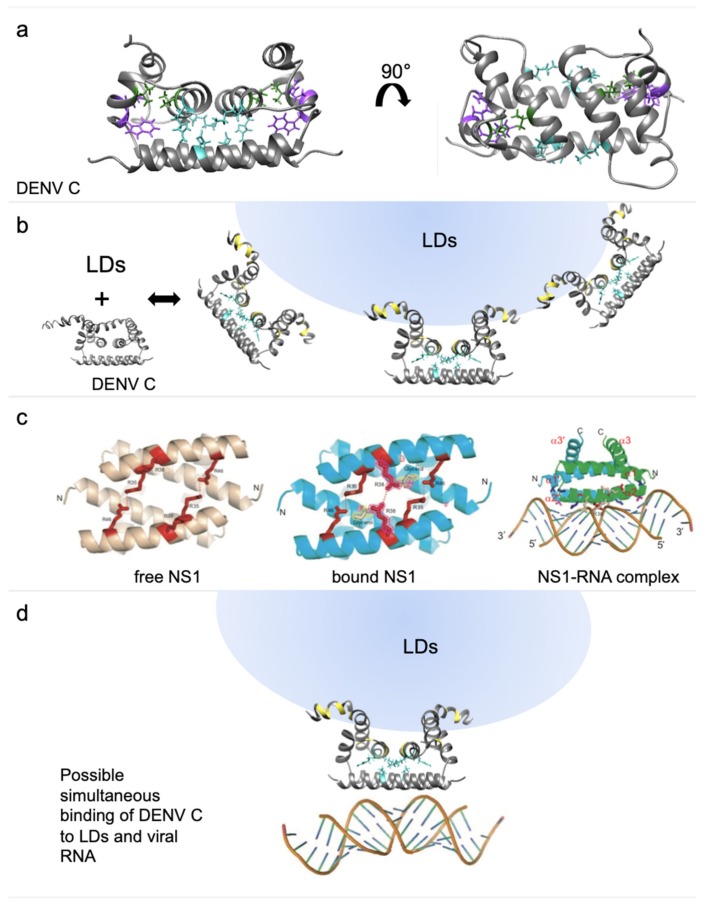
Protein structures of DENV C and influenza NS1. (**a**) DENV C structure from two different angles with the conserved residues R68 and W69 (purple) and the interface stabilizing residues V51 and I65 (green), as well as E87, R55 and K45, forming the salt bridge (cyan). (**b**) DENV C structure in a N-terminal region closed conformation and, next, in an open conformation with schematic binding of lipid droplets (LDs) and the affected amino acid residues (yellow). (**c**) The RNA-binding domain of NS1 protein from influenza A in a RNA-free (left) and RNA-bound state (middle and right), showing an organization similar to DENV C α4–α4′ region (adapted from Cheng et al., 2009 [44]). (**d**) DENV C with schematically bound to a LD and to RNA. DENV C amino acid residues affected by the binding to LDs are colored yellow, while a key internal salt bridge is shown in cyan. DENV C binding to host LDs may enable allosteric rearrangements (eventually involving the salt bridge), allowing a small conformational change in α4 side chains, namely the positively charged residues, prompting stable RNA-C protein binding.

These findings must also be considered in light of DENV C biologically relevant interactions with LDs [22] and RNA (Figure 7). DENV C experimental structure [12] contains three distinct structural regions [13]: a disordered N-terminal region (from the N-terminal up to residue R22), a flexible fold (residues V23 to L44, where α-helix 1 is located) and a conserved fold with helices α2, α3 and α4, containing the R68 and W69 amino acid residues, highly conserved among *Flavivirus* [12]. R68 terminates α3 helix, with its side chain pointing to the protein interior [12]. W69 locates at DENV C α4–α4′ interface, having a crucial role in the dimer structural stabilization [12]. Along with dimer structural stability, these interactions enable allosteric communication and movements between DENV C more hydrophobic section (α2–α2’dimer interface) and its remaining sections, namely the α4–α4′ region. Figure 7 displays this, in the context of the C protein biologically relevant interactions, as they are understood on the basis of recent studies [12,13,14,15,18,20,21,22,23,24].

Looking further, it is important to consider that the binding of DENV C to host LDs is mediated by both the N-terminal IDP region and the α2–α2′ interface [14]. V51 of α2 is affected by the interaction with LDs and stabilizes the dimer by contacting with α3 (I65). Another interaction via salt bridges, between α2 (K45 and R55′) and α4 (E87), stabilizes the homodimer (Figure 7a). The C protein binding to host LDs, which affects the α2–α2′, can lead to changes in the α4–α4′ structural arrangement (Figure 7b). To investigate this we searched for similar proteins. An RNA-binding protein with a two-helix domain similar to DENV C α4–α4′ was identified (Figure 7c), influenza A non-structural protein 1 (NS1, PDB ID: 2ZKO [44]). Influenza NS1 has interesting features: it accumulates in the nuclei of host cells after being translocated by importin α and β and works as a viral immuno-suppressor by weakening the host cell gene expression [45]. DENV C was also reported to have an importin α-like motif in the N-terminal [15,46]. Regarding the targets that may interact with importin α and be transported to the nucleus, they normally contain a nuclear localization sequence (NLS), consisting of a motif of at least 2 consecutive positively charged residues [47,48,49,50,51]. Some of these proteins contain 2 NLS motifs, with at least 8 (up to 40 or even more) residues in between, designated as a bipartite NLS motif [49,50,51]. Strikingly, *Flavivirus* C proteins have three motifs of two consecutive cationic residues in the N-terminal region and α1 domain, which could form a bipartite NLS. A bipartite NLS formed by the cationic residues before position 10 and at positions 17 and 18, with a spacer of 7 to 13 residues can occur. The other bipartite NLS possibility may be formed by residues at positions 17 and 18, and at positions 31 and 32, with 9 to 12 spacer residues. Possible bipartite NLS are also seen in the conserved fold region but its static nature precludes activity as NLS. If DENV C binds to importin α, it may act as a cargo protein to be transported to the nucleus. This could explain why has DENV C been found in the nucleus of DENV infected cells [46,52,53]. DENV C may directly bind importin β, given the similarities between the N-terminal region of DENV C and importin α [49]. This may allow it to disrupt the normal nuclear import/export system in DENV-infected cells. The conformational plasticity of the N-terminal and flexible fold regions is certainly compatible with interactions with importin(s). As the hypothesized bipartite NLS are conserved among *Flavivirus* C proteins, this may occur in other *Flavivirus*.

The C protein may act as an immuno-suppressor, similarly to influenza NS1, by interacting with importins α and/or importin β. Ivermectin, a specific inhibitor of importin α/β-mediated nuclear import, is able to inhibit HIV-1 and DENV replication [54]. The mechanism of DENV C inhibition might involve the C protein, specifically the intrinsically disordered N-terminal IDP region, which is similar to importin α disordered N-terminal region [15]. Moreover, influenza NS1 can counteract the RNA-activated protein kinase (PKR)-mediated antiviral response through a direct interaction with PKR [55]. Besides, influenza NS1 blocks interferon (IFN) regulatory factor 3 activation, which in turn prevents the induction of IFN-related genes [56]. DENV inhibits the IFN signaling pathway in a similar manner [57]. By its N-terminal region dsRNA-binding ability, influenza NS1 inhibits the nuclear export of mRNAs and modulates pre-mRNA splicing, suppressing antiviral response [44]. Similarities between DENV C and influenza NS1 also extend to the later ability to bind RNA (Figure 7c). Recognition of dsRNA is made by the influenza NS1 RNA-binding domain, which forms a homodimer [44]. Afterwards, a slight change in R38-R38′ orientation leads to anchoring the dsRNA to the protein by a hydrogen bond network to the protein [44]. One of the main functions of influenza NS1 binding to RNA is sequestering dsRNA from the 2′–5′ oligo(A) synthetase [58]. We propose that, as with influenza NS1, a small conformational change in DENV C α4–α4′ interface occurs after the contact of its α2–α2′ interface with LDs, modulated by transitions between alternative N-terminal “open” and “closed” conformations. Binding to LDs requires an open conformation (Figure 7d), decreasing the conformational variability and entropy of the C protein, which trigger the allosteric movements affecting the C-terminal α4–α4′. As with influenza NS1, the *Flavivirus* C protein would remain in the same overall fold, but a small opening of α4–α4′ would facilitate its binding to RNA.

The C-terminal is likely to be the crucial section for RNA binding given its similarity with influenza NS1 (Figure 7). Nevertheless, the N-terminal conformers must also be considered in the context of RNA binding (Figure 4). The A and D conformers allow RNA to be bound to the α4–α4′ interface and, simultaneously, to the N-terminal cationic amino acid residues. A–A′ and D–D′ conformations result in the possible binding of a single continuous portion of RNA to both the C-terminal α4–α4′ and the N-terminal IDP region, making the RNA more tightly bound. Moreover, the A–B’, B–B’ and B–C’ conformations would enable the protein to bind two distinct sections of the RNA, one bound to α4–α4′ and another to the N-terminal regions. That arrangement may allow to further compact the viral RNA. The N-terminal IDP region putative binding to RNA should not be disregarded given its positive net charge (+7). It compares very well with the C-terminal α-helical region net charge (+8 for a monomer, +16 for α4–α4′ dimer interface). Both may thus bind RNA due to, mostly, electrostatic forces. This IDP region can thus provide multi-functionality by several modes of binding and different ligands, enabled by alternative conformations. It must be stressed that this is not unlikely. Viral proteins tend to have IDP regions that increase their biological activity [59,60,61]. In a proteome as small as that of flaviviruses (10 proteins), IDP regions augment the number of ligands with which it can interact. Less structure often means more function. This is an increasingly hot topic of recent research, leading to design of algorithms to identify these regions [62,63]. Further analysis will help understand the interaction between DENV C and its ligands.

To conclude, the data imply a common structure and functions for mosquito-borne *Flavivirus* C proteins. Moreover, studying DENV C rotational diffusion and thermodynamics reveals a stable protein due to the conserved fold maintaining the homodimer structure. These findings apply to other *Flavivirus* C proteins, supporting a common mechanism for their biological activity. Such understanding of this key protein structure and dynamics properties may contribute to the future development of C protein-targeted drugs to impair dengue virus and other *Flavivirus* infections.

## 4. Materials and Methods

### 4.1. Materials

Chromatography columns HiTrap Heparin (1 and 5 mL), Sephadex S200 and the chromatography equipment AKTA-explorer were from GE Healthcare (Little Chalfont, UK). Sodium dodecyl sulphate-polyacrylamide gel electrophoresis (SDS-PAGE) reagents were from BioRad (Hercules, CA, USA). Unless otherwise stated, other chemicals were purchased from Sigma-Aldrich (St. Louis, MO, USA).

### 4.2. Flavivirus C Proteins Primary, Secondary and Tertiary Structural Predictions

For primary structure alignments we used the 16 non-DENV *Flavivirus* polyprotein sequences identified in reference [14], plus the four DENV reference sequences from NCBI, namely: DENV serotype 1, strain 45AZ5, NCBI ID NP_059433.1; DENV serotype 2, strain New Guinea C, NCBI ID NP_056776.2; DENV serotype 3, strain D3/H/IMTSSA-SRI/2000/1266, NCBI ID YP_001621843.1; and, DENV serotype 4, strain rDEN4, NCBI ID NP_073286.1. For the phylogenetic trees, both the entire polyproteins and the C protein regions were used. For the alignments and subsequent data analysis, the residues next to the NS2B-NS3 protease cleavage site [64,65] were excluded, leaving only the C protein sequences. Alignments and the derived phylogenetic trees were performed via Clustal Omega web tool (http://www.ebi.ac.uk/Tools/msa/clustalo/) [66,67].

Statistical comparison of the disordered N-terminal plus flexible fold regions with the conserved fold region of *Flavivirus* C proteins, for G and P content, as well as charged amino acid residues, was performed via a paired *t*-test, using GraphPad Prism v5 software. *p*-values were always lower than 0.001.

Predictions of hydrophobicity and α-helix propensity were done using ProtScale server (http://web.expasy.org/protscale/) [26,27], tertiary structure predictions were performed via I-TASSER server (http://zhanglab.ccmb.med.umich.edu/I-TASSER/) [28,29,30], following previous approaches [15]. Briefly, *Flavivirus* C protein sequences from our previous work were employed [14]. DENV and WNV (serotype Kunjin) C structures were excluded, not serving as templates for the tertiary structure prediction. ZIKV C protein structure was also not included, as it was not yet determined when the modeling was conducted. This avoids a bias towards known homologous protein structures. Five I-TASSER models were obtained for each C protein sequence. These were superimposed with DENV C experimental structure (PDB ID 1R6R, model 21) [12] after root-mean-square deviation (RMSD) minimization in UCSF Chimera v1.9 software [68]. Clusters were formed based on the visual similarity between predictions. The number of N-terminal amino acid residues with backbone clashes with the other monomer backbone was calculated for each model. In our previous work [15], a DENV C predicted structure was excluded from further analysis if it had 6 clashes or more, as it would not be viable as an homodimer [15]. Here we excluded models with more than 5 clashes (28 models rejected). These would preclude homodimer formation and, thus, were not considered in the clusters analysis (Table 1 excluded models column).

### 4.3. Structure Comparison Between DENV C and Influenza NS1

Protein structures coordinates were extracted from the Protein Data Bank (PDB, www.pdb.org). PDB identification codes are specified ahead after each protein name. The protein structures were superimposed through UCSF Chimera 1.13.1 software MatchMaker tool. After that, we carefully analyzed the superposition visually. Then, using the Match-Align tool of UCSF Chimera, which returns a sequence alignment based on the regions and taking into account the structure superimposition, we identified the residues simultaneously similar in structure and sequence. Protein structure figures were obtained using UCSF Chimera 1.13.1 version [68].

### 4.4. DENV C Recombinant Protein Production and Purification

Recombinant DENV C protein expression and purification was conducted based on previous approaches [13]. We used a pET-21a plasmid containing DENV serotype 2 strain New Guinea C capsid protein gene (encoding amino acid residues 1–100) [69]. The protein was expressed in *Escherichia coli* C41 and C43 bacteria grown in lysogeny broth (LB) medium. The only differences in the purification protocol are the abolition of the ammonium sulfate precipitation step and the addition of a size exclusion chromatography step (with Sephadex S200) after the heparin affinity column chromatography, using an AKTA chromatography equipment. The C protein was purified in a 55 mM KH_2_PO_4_, pH 6.0, 550 mM KCl. DENV C protein purified fractions were concentrated with Amicon Ultra-4 Centrifugal Filters of 3 or 10 kDa nominal cut-off, from Millipore (Billerica, MA, USA). Concentrated protein samples were stored at −80 °C. Protein samples quality was assessed by SDS-PAGE and matrix-assisted laser desorption/ionization, time-of-flight mass spectrometry (MALDI-TOF MS) analysis. Very low degradation and the highest peak consistent with the expected mass of the protein monomer (11765 Da).

### 4.5. Time-Resolved Fluorescence Anisotropy

Time-resolved fluorescence spectroscopy measurements were performed in a Life Spec II equipment with an EPLED-280 pulsed excitation light-emitting diode (LED) of 275 nm (Edinburgh Instruments, Livingston, UK), acquiring the emission at 350 nm. DENV C (monomer) concentration was 20 μM in 50 mM KH_2_PO_4_, 200 mM KCl, pH 6.0 or pH 7.5, with 550 μL total volume, in 0.5 cm × 0.5 cm quartz cuvettes. The instrument response function, $\mathrm{IRF}\left(t\right)$, was obtained with the same settings, except emission, which was at 280 nm, with a solution of polylatex beads of 60 nm diameter diluted in Mili-Q water. Measurements were performed at 22 °C. Time-resolved fluorescence intensity measurements with picosecond-resolution were obtained by the time-correlated single-photon timing (TCSPT) methodology [35]. Measurements were performed at constant time, with 15 min per decay, acquiring 2048 time points in a 50 ns window. Four intensity decays, $I\left(t\right)$, were acquired in each condition, with excitation/emission polarizers, respectively at vertical/vertical positions, $I_{\mathrm{VV}}\left(t\right)$, vertical/horizontal positions, $I_{\mathrm{VH}}\left(t\right)$, horizontal/vertical positions, $I_{\mathrm{HV}}\left(t\right)$, and horizontal/horizontal positions, $I_{\mathrm{HH}}\left(t\right)$. The instrumental *G*-factor was calculated as [35]:

(1) $$G=\frac{\int_{0}^{50}I_{\mathrm{HV}}\left(t\right)dt}{\int_{0}^{50}I_{\mathrm{HH}}\left(t\right)dt}$$

The *G*-factor value obtained was 1.61. The intensity decay with emission polarizer at the magic angle (~54.7°, with respect to the vertical excitation polarizer), $I_{m}\left(t\right)$, avoids the effects of anisotropy. It can be calculated easily [35]:

(2) $$I_{m}\left(t\right)=I_{\mathrm{VV}}\left(t\right)+2GI_{\mathrm{VH}}\left(t\right)$$

with $I_{\mathrm{VV}}\left(t\right)$ and $I_{\mathrm{VH}}\left(t\right)$ depending on the time-resolved fluorescence anisotropy, $r\left(t\right)$, as:

(3) $$I_{\mathrm{VV}}\left(t\right)=\frac{I_{m}\left(t\right)}{3}\left(1+2r\left(t\right)\right)$$

(4) $$I_{\mathrm{VH}}\left(t\right)=\frac{I_{m}\left(t\right)}{3G}\left(1-r\left(t\right)\right)$$

Thus, $I_{m}\left(t\right)$ was used to obtain the fluorescence lifetime components, $\tau_{i}$, and the respective amplitudes, $\alpha_{i}$, for the DENV C W69. $I_{m}\left(t\right)$ was described by a sum of three exponential terms:

(5) $$I_{m}\left(t\right)=\overset{3}{\underset{i=1}{\sum }}\alpha_{i}e^{\left(-\frac{t}{\tau_{i}}\right)}$$

where the index *i* represents each component of the fluorescence decay. For the fitting to the data, $\alpha_{i}$ and $\tau_{i}$ values were obtained by iteratively convoluting $I_{m}\left(t\right)$ with the $\mathrm{IRF}\left(t\right)$:

(6) $$I_{m}^{calc}\left(t\right)=I_{m}\left(t\right)⊗\mathrm{IRF}\left(t\right)$$

and fitting $I_{m}^{calc}\left(t\right)$ to the experimental data, $I_{m}^{exp}\left(t\right)$, using a non-linear least squares regression method. The usual statistical criteria, namely a reduced $\chi^{2}$ value bellow 1.3 and a random distribution of weighted residuals, were used to evaluate the goodness of the fits [35]. Data analysis was performed using the TRFA Data Processing Package v1.4 (Scientific Software Technologies Centre, Belarusian State University, Minsk, Belarus) which allows calculating automatically the standard error (SE) for each fitted parameter [35].

The time-resolved fluorescence anisotropy, $r\left(t\right)$, is calculated via $I_{\mathrm{VV}}\left(t\right)$, $I_{\mathrm{VH}}\left(t\right)$ and *G* via_ENREF_52:

(7) $$r\left(t\right)=\frac{I_{\mathrm{VV}}\left(t\right)-GI_{\mathrm{VH}}\left(t\right)}{I_{\mathrm{VV}}\left(t\right)+2GI_{\mathrm{VH}}\left(t\right)}$$

In this case, the obtained *r*(*t*) can be fitted to a single exponential decay [35]:

(8) $$r\left(t\right)=r_{0}e^{\left(-\frac{t}{\tau_{c}}\right)}$$

where $r_{0}$ is the anisotropy when *t*→0 and *τ_c_* is the rotational correlation time. The $r\left(t\right)$ decays were globally analyzed in TRFA Data Processing Package v1.4 maintaining the previously obtained α_i_ and *τ_i_* values constant, and convoluting Equations (3) and (4) with the respective $\mathrm{IRF}\left(t\right)$, analogously to the analysis of $I_{m}\left(t\right)$, using Equation (8) to fit $r\left(t\right)$. Values obtained for both pH conditions were considered statistically different if their 95% confidence intervals (~1.96 × SE) do not overlap (corresponding to *p* < 0.05).

### 4.6. Rotational Correlation Time Corrections

The *τ_c_* of a molecule in solution is related with the solution viscosity, *η*, the molecular hydrodynamic volume, V, the Boltzmann constant, $k_{B}$, and the absolute temperature, *T*, as [35,70]:

(9) $$\tau_{c}=\frac{\eta V}{k_{B}T}$$

Based on Equation (9), $\tau_{c}$ can be corrected for different temperatures, considering that the molecular volume does not change significantly in a small temperature interval (±5 °C; i.e., V and k_B_ are constants), using [70]:

(10) $$\frac{T_{a}\tau_{c,a}}{\eta_{a}}=\frac{T_{b}\tau_{c,b}}{\eta_{b}}\Leftrightarrow \tau_{c,b}=\tau_{c,a}\frac{\eta_{b}T_{a}}{\eta_{a}T_{b}}$$

where the indexes ‘a’ and ‘b’ represent a different condition of *T* and *η*, taking into account the variation of *η* with *T* [37]. The *η* values were assumed to be those of pure H_2_O or 10% D_2_O in the case of the corrections for the NMR-based values (those from the literature). In this way, Table 5 below shows the values employed on the calculations [37]:

### 4.7. Temperature Denaturation Measurements via Circular Dichroism (CD) Spectroscopy

Circular dichroism spectroscopy measurements were carried out in a JASCO J-815 (Tokyo, Japan), using 0.1 cm path length quartz cuvettes, data pitch of 0.5 nm, velocity of 200 nm/min, data integration time (DIT) of 1 s and performing 3 accumulations. Spectra were acquired in the far UV region, between 200 and 260 nm, with 1 nm bandwidth. The temperature was controlled by a JASCO PTC-423S/15 Peltier equipment. It was varied between 0 and 96 °C, in steps of 2 °C, increasing at a rate of 8 °C/min and waiting 100 s after crossing 5 times the target temperature, *T*. Then, the system was allowed, at least, 120 s to equilibrate (sufficient time for a stable CD signal). Before and after denaturation, spectra were acquired at 25 °C, to determine the reversibility of thermal denaturation. DENV C monomer concentration was 20 μM in 50 mM KH_2_PO_4_, 200 mM KCl, pH 6.0 or pH 7.5, with 220 μL of total volume. Spectra were smoothed through the means-movement method (using 7 points) and normalized to mean residue molar ellipticity, [θ] (in deg cm^2^ dmol^−1^ Res^−1^).

For the CD temperature denaturation data treatment, we assumed a dimer to monomer denaturation model [71,72,73] in which the folded dimer, F_2_, separates into unfolded monomers, U, in a single step described by reaction R1:

(R1) $$F_{2}\Leftrightarrow 2U$$

In this system, the total protein concentration, $\left[P_{m}\right]$, in monomer equivalents, is described as:

(11) $$\left[P_{m}\right]=2\left[F_{2}\right]+\left[U\right]$$

Hereafter, concentrations are treated as dimensionless, being divided by the standard concentration of 1 M, in order to be at standard thermodynamic conditions. The fractions of monomer in the folded, $f_{F}$, and unfolded, $f_{U}$, states are calculated by [71,72]:

(12) $$f_{F}=\frac{2\left[F_{2}\right]}{\left[P_{m}\right]}$$

(13) $$f_{U}=\frac{\left[U\right]}{\left[P_{m}\right]}$$

(14) $$f_{F}+f_{U}=1$$

and the concentrations of folded dimer and unfolded monomer can be written in terms of $f_{U}$:

(15) $$\left[U\right]=f_{U}\left[P_{m}\right]$$

(16) $$\left[F_{2}\right]=\frac{f_{F}\left[P_{m}\right]}{2}=\frac{\left(1-f_{U}\right)\left[P_{m}\right]}{2}$$

Then, the equilibrium constant, $K_{\mathrm{eq}}$, of R1 is defined in terms of $\left[U\right]$ and $\left[F_{2}\right]$, or $f_{U}$ and $\left[P_{m}\right]$:

(17) $$K_{\mathrm{eq}}=\frac{{\left[U\right]}^{2}}{\left[F_{2}\right]}=\frac{{\left(f_{U}\left[P_{m}\right]\right)}^{2}}{\left(1-f_{U}\right)\left[P_{m}\right]/2}=\frac{2\left[P_{m}\right]\times f_{U}{}^{2}}{\left(1-f_{U}\right)}$$

which can be solved in order to $f_{U}$, with the only solution in which $f_{U}\in \left[0;1\right]$ being:

(18) $$f_{U}=\frac{\sqrt{8\left[P_{m}\right]K_{\mathrm{eq}}+K_{\mathrm{eq}}{}^{2}}-K_{\mathrm{eq}}}{4\left[P_{m}\right]}$$

The [θ] signal as a function of temperature [71,72,74], ${\left[\theta \right]}_{T}$, can be described as a linear combination of the signal of the folded, ${\left[\theta \right]}_{T,F}$, and unfolded states, ${\left[\theta \right]}_{T,U}$, weighted by $f_{U}$:

(19) $${\left[\theta \right]}_{T}={\left[\theta \right]}_{T,F}\left(1-f_{U}\right)+{\left[\theta \right]}_{T,U}f_{U}$$

where ${\left[\theta \right]}_{T,F}$ and ${\left[\theta \right]}_{T,U}$ have a variation with *T* described here by a straight line (*i* can be F or U) [72,74]:

(20) $${\left[\theta \right]}_{T,i}=m_{i}\times T+{\left[\theta \right]}_{0,i}$$

Equation (19) can be re-written to evidence $f_{U}$ and then substitute it by Equation (18) [71,72]:

(21) $${\left[\theta \right]}_{T}={\left[\theta \right]}_{T,F}+\left({\left[\theta \right]}_{T,U}-{\left[\theta \right]}_{T,F}\right)\frac{\sqrt{8\left[P_{m}\right]K_{\mathrm{eq}}+K_{\mathrm{eq}}{}^{2}}-K_{\mathrm{eq}}}{4\left[P_{m}\right]}$$

$K_{\mathrm{eq}}$ can also be described by the standard Gibbs free-energy, $\Delta G^{{}^{\circ}}$, of the reaction R1:

(22) $$K_{\mathrm{eq}}=e^{-\frac{\Delta G^{{}^{\circ}}}{RT}}$$

where R is the rare gas constant and *T* is the absolute temperature. The $\Delta G^{{}^{\circ}}$ function used to fit the data contains both the enthalpic, $\Delta H^{{}^{\circ}}$, and entropic, $\Delta S^{{}^{\circ}}$, variations with temperature, which take into account $\Delta H^{{}^{\circ}}{}_{T_{m}^{{}^{\circ}}}$, the specific heat capacity at constant pressure, $\Delta C_{p}^{{}^{\circ}}$, and the standard conditions’ denaturation temperature, $T_{m}^{{}^{\circ}}$, according to [74]:

(23) $$\Delta G^{{}^{\circ}}=\Delta H^{{}^{\circ}}{}_{T_{m}^{{}^{\circ}}}\left(1-\frac{T}{T_{m}^{{}^{\circ}}}\right)-\Delta C_{p}^{{}^{\circ}}\left(T_{m}^{{}^{\circ}}-T+T\;\ln\left(\frac{T}{T_{m}^{{}^{\circ}}}\right)\right)$$

In our data, $\Delta C_{p}^{{}^{\circ}}$ was statistically equal to 0 and, thus, Equation (23) can be simplified to:

(24) $$\Delta G^{{}^{\circ}}=\Delta H^{{}^{\circ}}{}_{T_{m}^{{}^{\circ}}}\left(1-\frac{T}{T_{m}^{{}^{\circ}}}\right)$$

Then, Equation (21) was combined with Equations (20), (22) and (24), and fitted to the data using GraphPad Prism v5 software, via the non-linear least squares method, to extract both the $\Delta H^{{}^{\circ}}{}_{T_{m}^{{}^{\circ}}}$ and $T_{m}^{{}^{\circ}}$, along with the respective SE values. Afterwards, $\Delta S^{{}^{\circ}}{}_{T_{m}^{{}^{\circ}}}$ can be obtained, since $\Delta G^{{}^{\circ}}=\;0$ kJ mol^−1^ at $T_{m}^{{}^{\circ}}$, via the following Equation:

(25) $$\Delta H^{{}^{\circ}}{}_{T_{m}^{{}^{\circ}}}-T_{m}^{{}^{\circ}}\Delta S^{{}^{\circ}}{}_{T_{m}^{{}^{\circ}}}=0\overset{}{\Rightarrow }\Delta S^{{}^{\circ}}{}_{T_{m}^{{}^{\circ}}}=\frac{\Delta H^{{}^{\circ}}{}_{T_{m}^{{}^{\circ}}}}{T_{m}^{{}^{\circ}}}$$

The SE of $\Delta S^{{}^{\circ}}{}_{T_{m}^{{}^{\circ}}}$ was calculated based on $\Delta H^{{}^{\circ}}{}_{T_{m}^{{}^{\circ}}}$, $T_{m}^{{}^{\circ}}$, and the respective SE values:

(26) $${\mathrm{SE}}_{\Delta S^{{}^{\circ}}{}_{T_{m}^{{}^{\circ}}}}=\left|\frac{\Delta H^{{}^{\circ}}{}_{T_{m}^{{}^{\circ}}}}{T_{m}^{{}^{\circ}}}\right|\times \sqrt{{\left(\frac{{\mathrm{SE}}_{\Delta H^{{}^{\circ}}{}_{T_{m}^{{}^{\circ}}}}}{\Delta H^{{}^{\circ}}{}_{T_{m}^{{}^{\circ}}}}\right)}^{2}+{\left(\frac{{\mathrm{SE}}_{T_{m}^{{}^{\circ}}}}{T_{m}^{{}^{\circ}}}\right)}^{2}}$$

Interestingly, for a dimer to monomer denaturation, $K_{\mathrm{eq}}$ depends on $\left[P_{m}\right]$ and, consequently, $\Delta G^{{}^{\circ}}$ also depends on $\left[P_{m}\right]$. This implies that $\Delta G^{{}^{\circ}}=0$ at $T_{m}^{{}^{\circ}}$ ($T_{m}$ value estimated if $\left[P_{m}\right]=1M$), which is considerably higher than the observed $T_{m}$ (that occurs when $f_{U}=0.5$). The dependence of $T_{m}$ with $\left[P_{m}\right]$ is [72]:

(27) $$\Delta G^{{}^{\circ}}{}_{f_{U}=0.5}=-RT_{m}\ln\left(\left[P_{m}\right]\right)\overset{}{\Rightarrow }T_{m}=\frac{\Delta G^{{}^{\circ}}{}_{f_{U}=0.5}}{-R\ln\left(\left[P_{m}\right]\right)}$$

(28) $$T_{m}=\frac{\Delta H^{{}^{\circ}}{}_{T_{m}^{{}^{\circ}}}}{\Delta S^{{}^{\circ}}{}_{T_{m}^{{}^{\circ}}}-R\ln\left(\left[P_{m}\right]\right)}$$

The SE of $T_{m}$ was based on the percentual SE value of $T_{m}^{{}^{\circ}}$.

Values obtained for both pH conditions were statistically evaluated via F-tests to compare two possible fits, one assuming a given parameter as being different for the distinct data sets, and another assuming that parameter to be equal between data sets (while maintaining the other parameters different). No statistically significant difference (*p* < 0.05) was observed.

## Figures and Tables

**Table 1:** Distribution of the I-TASSER predicted models through the four clusters.

Protein	Cluster A	Cluster B	Cluster C	Cluster D	Excluded
ALFV C	1	0	1	0	3
AROAV C	1	1	1	0	2
BAGV C	1	0	2	1	1
DENV C	1	2	1	0	1
IGUV C	1	2	1	0	1
ILHV C	0	2	2	0	1
JEV C	1	1	0	1	2
KEDV C	0	1	1	1	2
KOKV C	1	0	1	1	2
MVEV C	0	2	1	0	2
ROCV C	1	0	1	2	1
SLEV C	1	0	2	0	2
SPOV C	1	2	1	0	1
USUV C	1	0	2	0	2
WNV C	1	0	1	1	2
ZIKV C	0	1	0	1	3
**Total**	**12**	**14**	**18**	**8**	**28**

**Table 2:** Fitting parameters of DENV C time-resolved fluorescence anisotropy data analysis. Parameters obtained from fitting Equations (5) and (8) to the data of Figure 5. Values are average (±% standard error, SE). * Statistically significant differences (*p* < 0.05) between the values obtained for the two pH values tested.

Parameter	pH 6.0	pH 7.5
*τ*_1_ (ns) *	0.209 (± 3.9%)	0.520 (± 4.0%)
*τ*_2_ (ns)	3.106 (± 0.4%)	3.108 (± 0.9%)
*τ*_3_ (ns) *	6.328 (± 0.4%)	6.506 (± 0.4%)
α_1_ *	0.275 (± 0.7%)	0.178 (± 3.4%)
α_2_ *	0.315 (± 0.9%)	0.385 (± 0.4%)
α_3_ *	0.410 (± 0.4%)	0.437 (± 0.4%)
*τ_c_* (ns) *	16.46 (± 2.9%)	16.41 (± 3.4%)
*r*_0_	0.130 (± 0.8%)	0.131 (± 1.1%)

**Table 3:** Comparing DENV C *τ_c_* values (*τ_c_* at 25 °C in H_2_O were calculated using Equation (10)).

*τ_c_* (ns) at *T*	*T* (°C)	*τ_c_* (ns) at 25 °C in H_2_O	Method	Source
16.4 ± 0.5	22	15.2 ± 0.5	Time-resolved fluorescence anisotropy	This work
13.0	27	13.4	Overall NMR relaxation analysis	Jones et al., 2003 [16]
14.1	20	12.0	*τ_c_* (ns) ≈ 0.6×MW (kDa)	Rossi et al., 2010 [36]

**Table 4:** Fitting parameters of DENV C temperature denaturation CD data. Parameters were estimated by fitting Equation (21) (combined with Equations (20), (22), (24) and (28)) to the data. *T*_m_ is the experimentally observed melting temperature (represented by the vertical lines in Figure 6c). Estimations are average ± SE. There were no significant variations between the two pH values tested (*p* < 0.05).

Parameter	pH 6.0	pH 7.5
$T_{m}$ (°C)	70.02 ± 0.63	69.03 ± 0.65
$T_{m}^{{}^{\circ}}$ (°C)	88.26 ± 0.80	88.80 ± 0.83
$\Delta H^{{}^{\circ}}{}_{T_{m}^{{}^{\circ}}}$ (kJ mol^−1^)	612 ± 26	564 ± 23
$\Delta S^{{}^{\circ}}{}_{T_{m}^{{}^{\circ}}}$ (kJ mol^−1^ K^−1^)	1.693 ± 0.073	1.557 ± 0.065

**Table 5:** Values for *η* employed in this work, derived from the references and Equations above.

T (°C)	*η* in H_2_O (cP)	*η* in 10% D_2_O (cP)	$\frac{\eta_{b}T_{a}}{\eta_{a}T_{b}}\;\mathrm{in}H_{2}O$	$\frac{\eta_{b}T_{a}}{\eta_{a}T_{b}}\;\mathrm{in}10\%D_{2}O$
20	1.002	1.027	0.8736	0.8523
22	0.955	0.978	0.9231	0.9012
25	0.890	0.911	1	0.9770
27	0.851	0.871	1.0530	1.0293

## Abbreviations

ALFV	Alfuy virus
APOE	Apolipoprotein E
AROAV	Aroa virus
BAGV	Bagaza virus
C protein	Capsid protein
CD	Circular dichroism
DENV	Dengue virus
ICTV	International Committee on Taxonomy of Viruses
IDP	Intrinsically disordered protein
IFN	Interferon
IGUV	Iguape virus
ILHV	Ilheus virus
JEV	Japanese encephalitis virus
KEDV	Kedougou virus
KOKV	Kokobera virus
LDs	Lipid droplets
MVEV	Murray Valley encephalitis virus
NS1	Non-structural protein 1 from influenza virus A
PDB	Protein Data Bank
pep14-23	Inhibitor peptide pep14-23 (amino acid sequence NMLKRARNRV)
PLIN3	Perilipin 3
ROCV	Rocio virus
SLEV	Saint Louis encephalitis virus
SPOV	Spondweni virus
USUV	Usutu virus
VLDL	Very low-density lipoproteins
WNV	West Nile virus
WNV-K	WNV serotype Kunjin
YFV	Yellow fever virus
ZIKV	Zika virus
